# Effect of Age-Hardening Temperature on Mechanical and Wear Behavior of Furnace-Cooled Al7075-Tungsten Carbide Composite

**DOI:** 10.3390/ma15155344

**Published:** 2022-08-03

**Authors:** Srinivasan Rajaram, Thirugnanam Subbiah, Parammasivam Kanjikovil Mahali, Muthuramalingam Thangaraj

**Affiliations:** 1 Department of Mechanical Engineering, SRM Valliammai Engineering College, Chennai 603203, India; thirugnanams.mech@valliammai.co.in; 2 Department of Aerospace Engineering, Madras Institute of Technology Campus, Anna University, Chennai 600044, India; mparams@annauniv.edu; 3 Department of Mechatronics Engineering, SRM Institute of Science and Technology, Kattankulathur 603203, India; muthu1060@gmail.com

**Keywords:** Al7075, WC, age hardening, temperature, fractography, wear

## Abstract

In this study, aluminum alloy (Al7075) composites with a 4% weight fraction of tungsten carbide (WC) were manufactured using a stir casting process and the developed composites were subjected to various ageing temperatures. An attempt has been made to predict the age-hardening temperature with the enhanced mechanical and wear properties of Al7075-WC. The result shows that the composite specimen aged at 250 °C offered maximum tensile strength and the Brinell hardness number was increased by 37.1% and 50.5%, respectively; the maximum impact energy was observed to be 92.2% for the 450 °C aged composites, compared to the non-aged Al7075-WC composites. The strength properties of the Al7075-WC composite decreased to 30.86%, 4.7%, and 24.9% when the composite specimens aged at 350 °C. The mechanical properties of the Al7075-WC composite were increased at the age-hardening temperatures from 150 °C to 250 °C and decreased from 250 °C to 350 °C. The wear testing pin-on-disc setup utilized to determine the wear characteristics of the prepared MMC with wear parameters of load and sliding distance and the wear resistance of the composite specimens increased due to ageing. The fractography analysis of the composite samples carried out by scanning electron microscope (SEM) images revealed that the fracture of the composite during the tensile test is a mixture of ductile and brittle modes.

## 1. Introduction

Aluminum matrix composites are widely used in many engineering applications because of their superior properties, which include being light weight, their high strength, and better corrosion resistance. By increasing the addition of weight percentage of ceramic particles in the aluminum matrix, this improves its hardness, tensile, compressive, and flexural strengths [1,2,3,4]. Due to the heat-treatment process, the formation of precipitates inside the AA6061 improves their mechanical properties [5]. Heat treatment of AA7085 improves corrosion resistance and mechanical properties [6]. The various heat treatments of AA6070 offer significant variations in the mechanical properties of the alloy, without altering the grain shape [7]. Large dimples characterize the fracture toughness of AA7085 under heat treatment, and the fracture mode is mixed with excessive transgranular failure and intergranular failure, which leads to the improved mechanical properties of composite [8]. After the heat treatment process, the improvement in residual stresses increases the fatigue strength of the AA2124 composites [9]. The tribological behavior of AMCs can be improved using heat treatment, as it relaxes the stresses and improves the dislocation rate [10]. For the Ti/TiB2 composite developed by the sintering and multiaxial forging process, the hardness and wear of the composite increased with the increasing addition of TiB, whereas the hardness of forged composite decreased compared to the sintered composites [11]. The Al-CNT composite developed by solution high energy ball milling, ball milling and mechanical coating techniques concluded that the strength of the composite increased by CNTs, regardless of its length. The mechanical coating and solution ball milling method were more effective methods of fabrication of the composite than the energy ball milling method [12]. The spark plasma sintering technique was used to fabricate the Ti-TiB composite and reported that the tensile strength of the composite decreased due to the higher ductility that occurred while increasing the temperature greater than 500 °C [13]. Titanium was used as the matrix material and nanosized whisker of TiB as the reinforcement to develop the composite by the plasma sintering process. The TiB whisker plays a vital role in enhancing the properties of the titanium alloy matrix and better strength was obtained for the 100 nm diameter size of the TiB whisker [14]. The composite was developed by the spark plasma sintering process using Ti, Mo and TiB powder, which reported that under high pressure torsion, the micro hardness of the composite specimen is higher nearby the edge than that of the center and precipitation hardening is also important process to enhance the hardness of composite [15]. The Ti-15 Mo/TiB composite fabricated by the SPS method and the developed composite were subjected to the hot rolling process, reported to marginally increase the tensile elongation from 2% to 12.5% at the same temperature due to the hot rolling of the composite, compared with the cast composite [16]. Microhardness of the Ti-15 Mo/TiB metal-matrix composite was carried out after the laser beam welding at different preheating temperatures and concluded that the peak microhardness value of the composite was obtained at the preheating temperature of 200 °C [17]. Titanium-titanium boride composites developed by the different fabrication method demonstrated that the toughness and wear resistance of the composite is dependent on the volume fraction of the reinforcement addition to the matrix [18]. Maximum tensile strength of the Ti/TiB metal-matrix composite was reached at 500 °C and beyond this, the temperature strength decreased, which is fabricated through spark plasma sintering process [19].

The Al7075 alloy is mostly preferred for high-strength applications, including structural parts of marine, aerospace, and automobiles and prosthetic devices, due to the presence of zinc and a small amount of magnesium, which improves the strength and resistance to temperature changes when compared to other aluminum alloy composites [20,21]. Generally, the MMCs have excellent properties, including being lightweight, their higher elasticity, higher strength, etc., which have replaced conventional metallic materials in automobile and aerospace industries. These particles make the aluminum matrix plastically constrained, which improves its high-temperature properties and offers superior mechanical properties and wear resistance [22,23]. Compared with the other MMCs, the Al7075-tungsten carbide composite has a high density and its mechanical, electrical, and wear behaviors are better [24,25]. Thus, to improve Al-7075, which is lightweight and involves robust manufacturing, the tungsten carbide reinforcement is selected for this research work. The composite subjected to age-hardening treatment was found with a 5% increase in tensile strength [26]. The stress relaxation behavior of AA7075 annealed at 413 °C for two and half hours was semi-logarithmic and accompanied the hardening process [27]. AA7075 subjected to a multi-step heat treatment process offers low spring back, aggregate exfoliation corrosion resistance and tensile strength because of the dislocation recovery and greater strain relaxation at higher temperatures [28]. The Al-Cu-Mg/Nano SiCp composite fabricated by the stir casting technique with 4% Nano SiCp reinforcement and subjected to age hardening with different temperatures, such as 175 °C, 200 °C and 225 °C, was reported as a composite aged at 225 °C that exhibited a low wear rate on both counter surfaces [29]. It was found that the addition of tungsten carbide reinforcement particles to an aluminum matrix means that it exhibits superior mechanical properties to silicon carbide [30]. The impact strength is high at 2% of the reinforcement, due to the reduction in dimples voids compared to the further addition of WC reinforcements and base alloys [31]. It was inferred that interfacial bonding among the AA5083 matrix and WC particles was strong and showed no interfacial reaction to weakening the interface, which leads to improved composite properties [32]. The diffusion of WC in the cobalt and iron matrix was in dynamic equilibrium due to the absence of new phases [33]. So, tungsten carbide is preferred over the other reinforcement particles. The wear rate decreases as the hardness of the material increases. The wear volume loss increases as the applied load or sliding speed increases [34]. The hybrid composite was made with silicon carbide and rice husk ash as the reinforcement. It was concluded that with an increase in the sliding distance and load, the wear loss of the hybrid composite increased, and resistance to wear increased, while increasing the addition of RHA reinforcement up to 10% and beyond that, it decreased [35,36]. When the composite specimens were subjected to higher loads, it was found that a significant quantity of material was removed from the composite surface, which underwent a high level of plastic deformation. The results demonstrate that the composite has a low wear resistance [37]. The fabricated composites were age hardened at 50, 100, 150, 200 and 250 °C for 1 h and reported that an increase in the age-hardening temperature meant it exhibited high hardness and low wear up to 200 °C and beyond that, the strength was decreased [38]. Two different melting temperature were used to melt the composite (680 °C and 850 °C), stir casting procedures were employed to fabricate the Al-SiC composites with stirring times of 0, 2, and 4 min and reported that higher stirring temperatures improved the distribution of the reinforcement in the matrix [39]. The developed composites were solutionized in ice, water and oil medium, and subsequently aged hardened at 175 °C for 10 h, concluding that ice quenched composites exhibit high hardness and tensile strength compared with water and oil quenched composites [40]. Mg/B_4_C composites were developed with different boron carbide reinforcement weight percentages of 3, 6, 9, and 12. The composite was not softened under the same condition with the high wear resistance of the magnesium alloy [41]. For the Al-SiC composite, the maximum removal of material from the material surface is an alkaline environment, followed by aqueous medium and dry sliding environments [42]. An aluminum 375-SiC dual size reinforcement composite was prepared by the stir casting route, the composites were artificially aged at 160 °C, 180 °C, 200 °C and concluded that the tensile strength and hardness value increased up to 180 °C and beyond that, the strength of the composites decreased [43]. When the composites are aged, the strength properties are reported to be increased when compared to the base aluminum matrix [44]. Al7075-Albite particulate composites were quenched in ice, water, and air medium after the quenching composites were artificially aged at 120 °C and it was concluded that the peak hardness value is reached when the composite is quenched in ice at 6 h of aging [45]. The mass loss was decreased by increasing the addition of weight percentage of tungsten carbide [46]. Two different types of hybrid composite of AA5052-WC-graphite and AA5052-SiC-graphite, formed through the stir casting process by increasing the weight percentage (5, 10, 15%) addition of WC and SiC with 4% wt of graphite, reported that the tungsten carbide hybrid composite exhibited higher strength when compared with the silicon carbide hybrid composite and also the strength of the hybrid composite increased, while increasing the reinforcements [47]. The LM-25/tungsten carbide composite was developed and subjected to heat treatment and rapidly quenched and concluded that the properties of the composite increased during the ageing of the composite [48]. The metal matrix composite fabricated by the stir casting process by varying the weight of WC content (1.5, 3, 4.5 and 6 wt.%) with aluminum 7075 reported that the tensile strength and hardness increased and the impact energy of the composite was decreased with the increasing addition of the reinforcement particle to the aluminum matrix [49].

The majority of researchers are focused on evaluating the mechanical behavior of metal matrix composites through an ageing process in which the composites are exposed to various ageing time periods under constant temperatures and age-hardened composites are cooled using various cooling medium other than a furnace. According to the literature review, there is no work carried out on aluminum tungsten carbide composites subjected to the age-hardening process. The uniqueness of this research is to study the mechanical and wear behavior of Al7075-WC composites aged at various temperatures at constant time periods and then the aged composites are cooled in an electrical furnace. The objective of this research was to use a heat treatment process to improve the mechanical and tribological properties of Al7075 4% weight fraction of tungsten carbide-reinforced composites [50], used for brake pads in automobile industries. These developed composites were subjected to artificial ageing temperatures of 0 °C (non-ageing), 150 °C, 250 °C, 350 °C, and 450 °C for 2 h and after that, the aged composites were cooled in the furnace. Tensile strength, hardness, impact strength, and wear resistance were tested, as well as the composite failure mechanism after the tensile test was explored. X-ray diffraction, scanning electron microscope (SEM), and optical microscope were used to examine the composites’ composition and microstructure. The mechanical and wear behavior with the function of the age-hardening temperature were investigated and reported.

## 2. Materials and Methods

### 2.1. Fabrication of Composites

Aluminum grade Al7075 was used as the base matrix material for this research work. It was purchased from Perfect metal works, Bangalore, Karnataka, India. The aluminum alloy 7075 contains zinc as the primary alloying element and Table 1 shows the chemical composition of Al-7075 as a percentage of the weight fractions. The reinforcement of tungsten carbide (WC) for this research work was purchased from Rapicut Carbides Limited, Chennai, India, with an average particle size of 3–5 µm. The important reason why we used the tungsten carbide as the reinforcement, which comprises equal parts of carbon and tungsten atoms, is because it maintains its toughness and strength at different temperature ranges. The major chemical and physical properties of tungsten carbide are listed in Table 2 and Table 3. The metal matrix composite is prepared by the stir casting method. At the first stage of fabrication of the composite, the Al7075 aluminum alloy is charged into the graphite crucible and heated to around 750 °C, until the entire matrix material in the crucible has melted completely, before adding the tungsten carbide (WC) reinforcement to the molten aluminum matrix, which is preheated to 250 °C for 20 min in the furnace to remove moisture in the reinforcement. The degassing tablet of nitral was introduced after the matrix metal had completely melted to significantly reduce porosity during composite production. A stirrer with a blade angle of 45° was gently placed into the melt to stir the molten metal at a speed of 350 rpm, which was controlled by a regulator connected to the furnace. To obtain uniform distribution of WC in the matrix metal, the warmed reinforced particle of tungsten carbide was added to the Al7075 molten metal at a steady rate and stirred for 3 min to achieve uniform distribution of WC in the composite [51]. After complete stirring of the molten mixture, the Al7075-WC mixture was poured into a warmed square-shape mold, with dimensions of 120 × 120 × 25 mm, after adequate mixing. The molten mixture Al7075-WC was poured into the mold and allowed to cool at an ambient temperature. After complete cooling of the mold, the parent composite materials were removed from the mold. The specimens for tensile strength, hardness, impact strength and wear test were prepared from the parent cast composite based on the ASTM standard. The ageing process of AA7075-WC includes solutionizing and artificial ageing. The developed parent composite was heated in an electrical furnace at 520 ± 5 °C for 3 h, and quenched in room temperature water, then artificially aged at 150 °C, 250 °C, 350 °C, and 450 °C for 2 h. The age-hardened composite was subsequently cooled in the electrical furnace.

### 2.2. Measurement of Performance Measures

**Figure 1:**
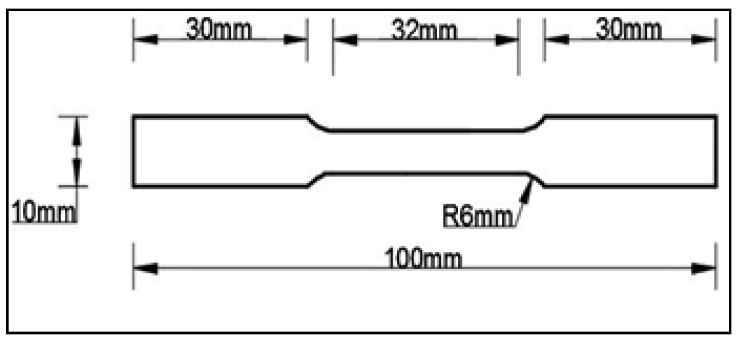
Specification of tensile test specimen.

Tensile test specimens were made from the parent composite for tensile testing, according to ASTM-E8 and dimensional specifications of tensile samples, as shown in Figure 1, for all age-hardening temperatures. A computer servo controlled ball screw operated UTM tensometer set up was used to carry out the tensile test of the Al7075-WC composite. At room temperature (30–32 °C), the Brinell hardness of the composite was measured using a Brinell hardness tester with a steel ball indenter. The dimensional standard of Al7075-WC for hardness testing is 10 × 10 × 10 mm size specimens, which were prepared from the parent material for different ageing temperatures by applying a load of 500 g to the composites for 30 s. The hardness values were measured from three different locations of all the aged composites and an average of the measured hardness value for each aging temperature was taken as the hardness of the AA7075-WC composites. The Charpy impact tests were performed at room temperature and used as impact testing equipment with a specimen dimension of 55 × 10 × 10 mm and a notch depth of 2 mm. There were three composite samples that were tested for each artificially aged temperature and the average value for impact strength was taken. A wear test of the Al7075-WC composite was carried out to assess the wear performance of the composite specimen and the specimen was machined as per ASTM G99 from the parent composite.

**Figure 2:**
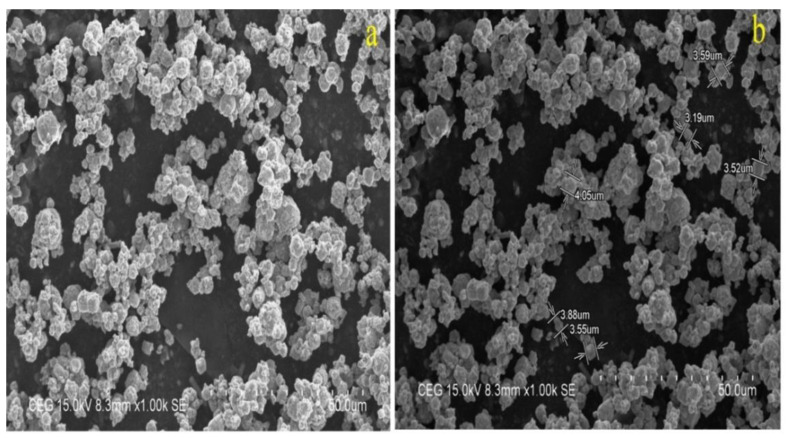
(**a**) Tungsten carbide SEM morphological image; (**b**) size of the reinforcement.

The computerized pin-on-disc test setup was used to evaluate the dry sliding wear behavior of the Al7075-WC composite and the size of the composite used for the wear test was 8 mm diameter with 30 ± 1 mm length. The counter face of the wear disc machine was made up of EN-31 steel and the speed of the rotating disc used in the conduction test was 600 rpm, with sliding distances of 1000 m, 1500 m and 2000 m for all composite samples. SEM analysis was used to investigate the microstructures of the MMC composites. The elemental analysis and morphological characteristics of the composites were examined using scanning electron microscopy (SEM: JEOL JAPAN, Jeol-6490 JED-2300) with energy dispersive X-ray spectroscopy (EDX). To evaluate the effect of heat treatment on the characteristics of the composites, X-ray diffraction analysis was conducted. Figure 2a,b show the SEM morphological images and sizes of tungsten carbide (WC) reinforcement, respectively.

## 3. Results and Discussion

### 3.1. Microstructure Analysis Using SEM

**Figure 3:**
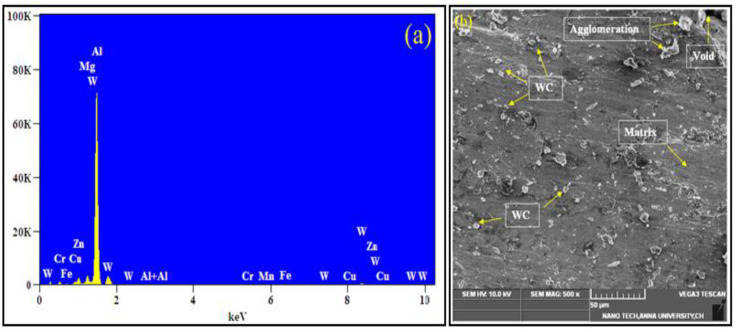

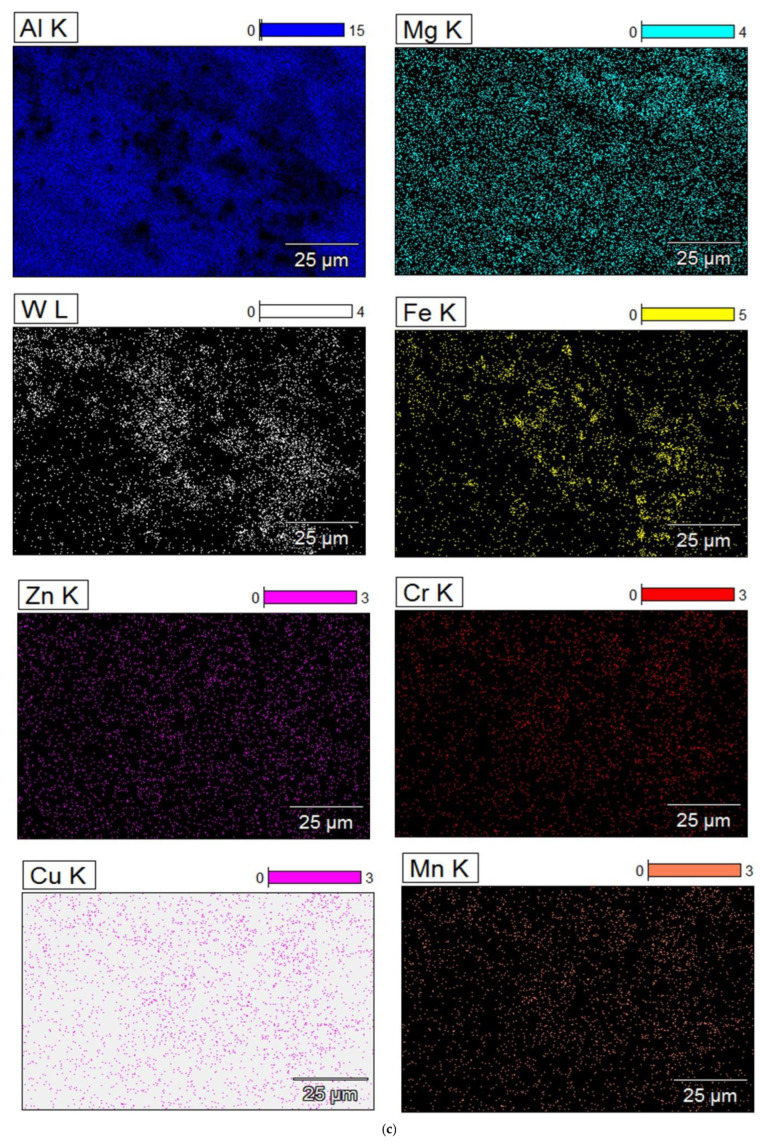
(**a**) EDAX profile of composite. (**b**) Distribution of WC in Al7075-WC composites. (**c**) SEM-MAP distribution alloying element on AA7075-WC composites.

The mechanical and tribological behavior of the Al7075-WC composite were examined in detail at various ageing temperatures, including non-aged, 150 °C, 250 °C, 350 °C, and 450 °C for 2 h and all the age-hardened composite samples were subsequently cooled in the furnace. The presence of WC reinforcement and elements in the produced Al7075-WC composite of W, Cr, Mg, Cu, Fe, Zn, Al, and Mn were identified in the elemental analysis presented in Figure 3a, and the SEM images with EDX of MMC confirmed that the above-mentioned elements were present in the composite. In the SEM microstructure analysis of MMC, it clearly showed the uniform distribution of tungsten carbide in the aluminum matrix, as shown in Figure 3b.

Figure 3c depicts the MAP analysis of an Al7075-WC composite to identify the distribution of alloying elements in the composite structure. The result shows that the elements present in the Al7075-WC composites were distributed homogeneously. It is observed that the aluminum covers the entire surface and its alloying elements were distributed uniformly. It is also observed that the reinforcement was distributed evenly over the composite.

### 3.2. Microstructure Analysis Using SEM

**Figure 4:**
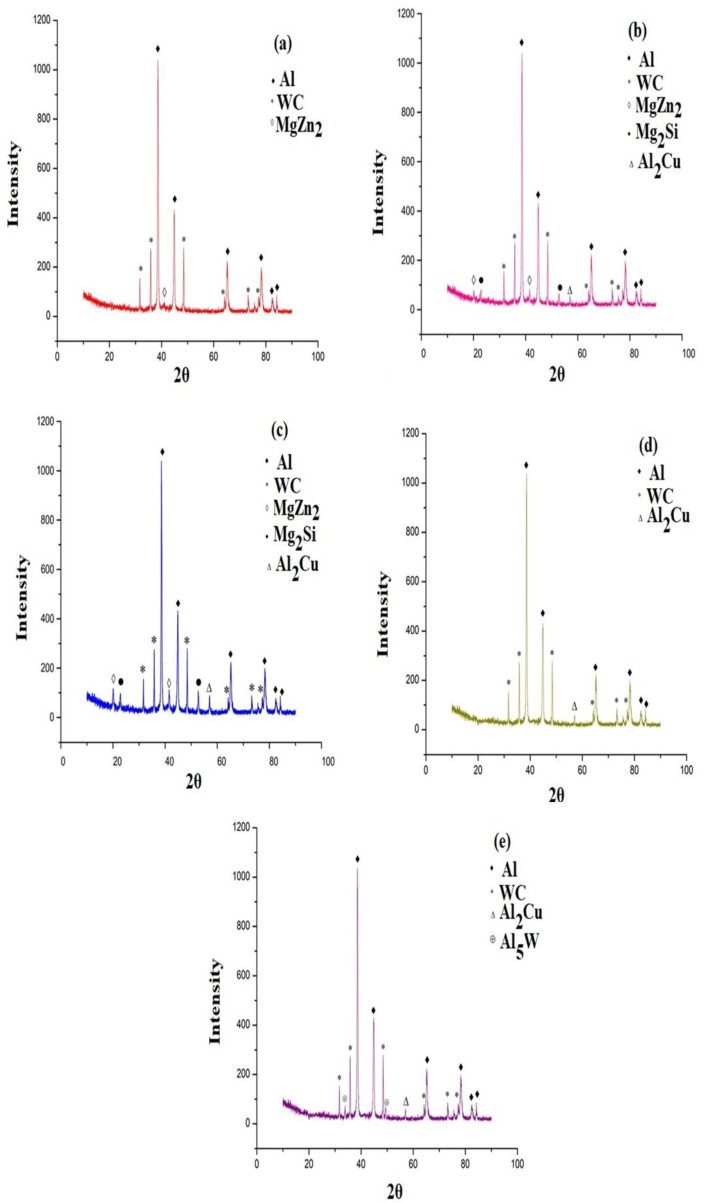
X-Ray diffraction analysis of Al7075-WC composite of (**a**) non-aged, (**b**) aged at 150 °C, (**c**) 250 °C, (**d**) 350 °C; (**e**) 450 °C.

The XRD patterns were observed and analyzed using Al7075-WC age-hardened samples with 5 mm length, 5 mm breath, and 5 mm thickness. Figure 4a–e show different levels of peaks that were obtained in the Al7075-WC composite artificially aged with different ageing temperatures, the non-aged composite and the composites aged at 150 °C, aged at 250 °C, aged at 350 °C and aged at 450 °C. In all of the XRD patterns of the artificially aged composites, the longest peaks of aluminum and peaks of WC were found. The patterns of the composite aged at 150 °C and 250 °C show that the formation of the secondary phase of Al and Mg precipitates (Al_2_Cu, Mg_2_Si and MgZn_2_) increases while increasing the age-hardening temperature, as shown in Figure 4b,c, whereas for the composite aged at 350 °C, the precipitates of Mg and Al decreased and the peaks were relatively too small, as compared with other aged composites, due to the dissolving of the elements present in the composite, as shown in Figure 4d.

The X-ray diffraction peaks confirm the formation of precipitate during the ageing of the composite, including Al_2_Cu, Mg_2_Si and MgZn_2_, which were formed due to the reaction between the compositional elements present in the aluminum alloy during the artificial ageing of the Al7075-WC composite. The intermetallic compound of Al_5_W was observed only in the composite aged at 450 °C and reported that the presence of the Al_5_W compound can contribute towards the enhancement of the mechanical properties of the Al7075-WC composite, which includes strength and thermal stability of the materials, as shown in Figure 4e. The same phenomena for the tungsten aluminide phase were identified and reported in the XRD analysis by Gurpreet Singh et al. [52].

### 3.3. Tensile, Hardness and Impact Behavior of Composites

**Figure 5:**
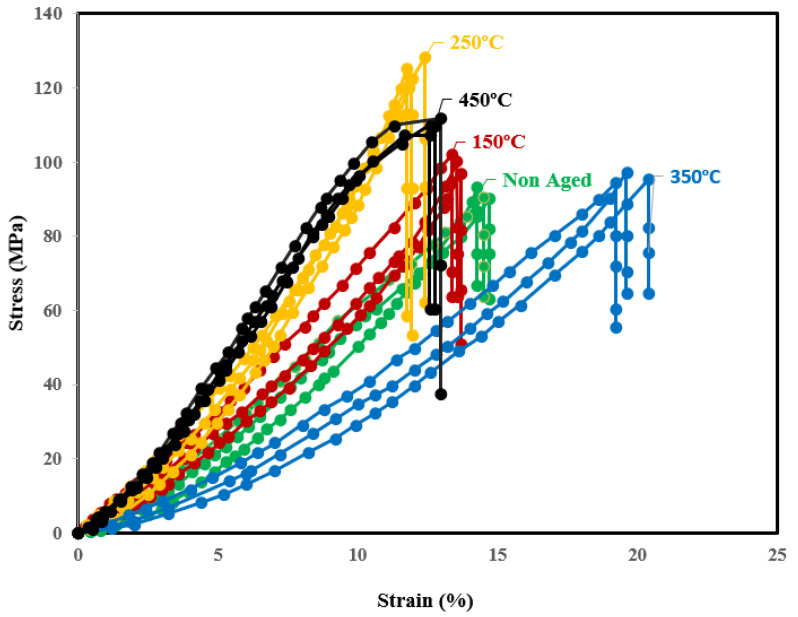
Stress strain curve of non-aged and aged composites at 150, 250, 350 and 450 °C.

**Figure 6:**
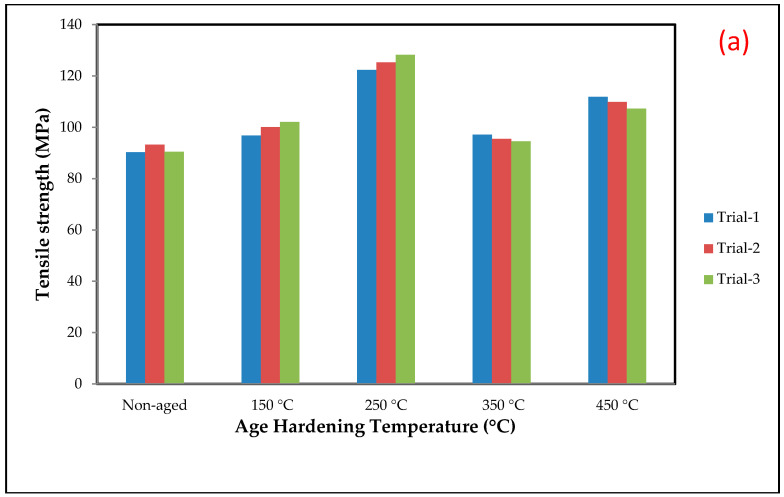

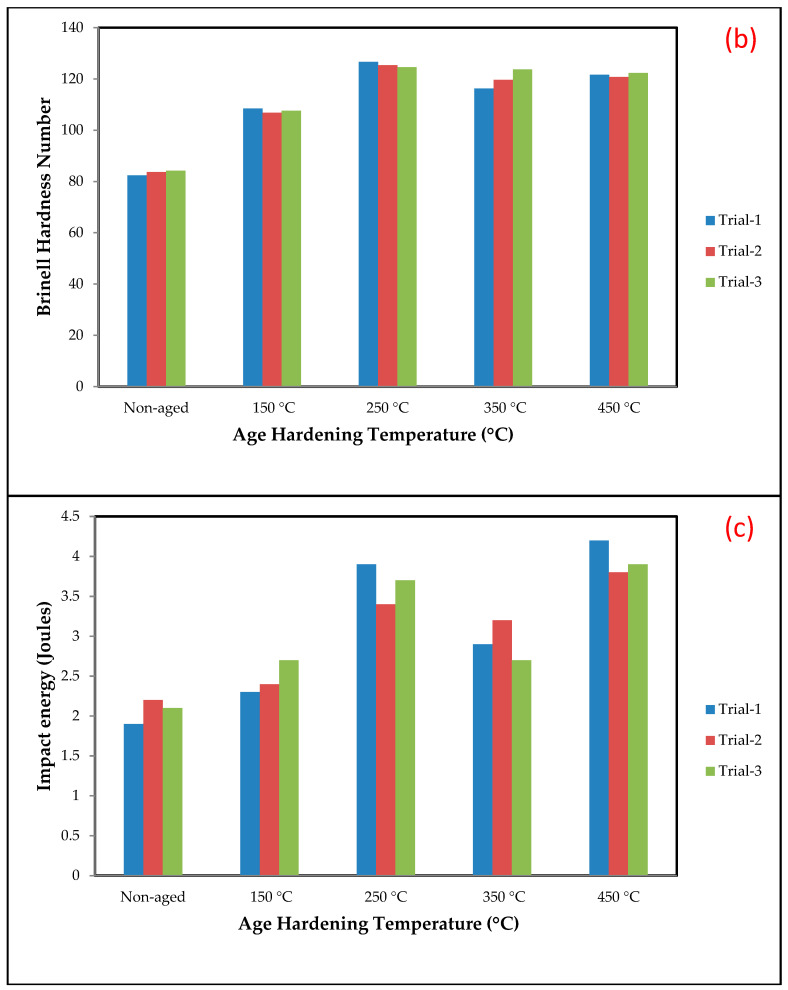
(**a**) Tensile strength, (**b**) Brinell hardness number, (**c**) impact energy of non-aged and aged at 150, 250, 350 and 450 °C composite specimens.

**Figure 7:**
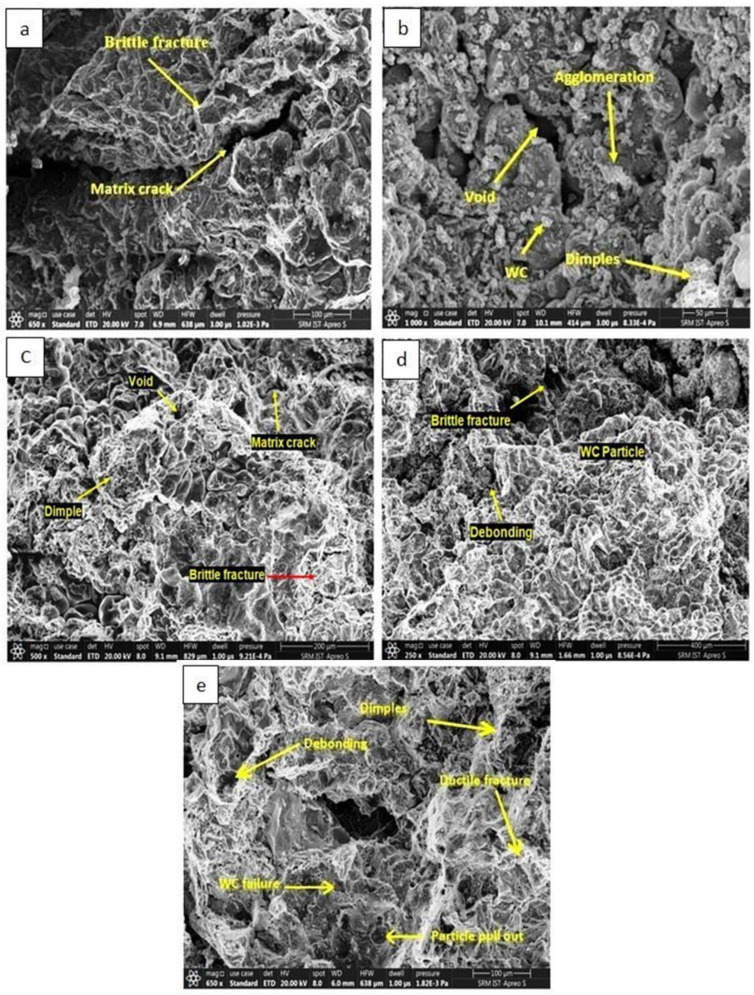
SEM Fractography image of (**a**) non aged and aged at (**b**) 150 °C, (**c**) 250°, (**d**) 350 °C and (**e**) 450 °C specimen.

**Figure 8:**
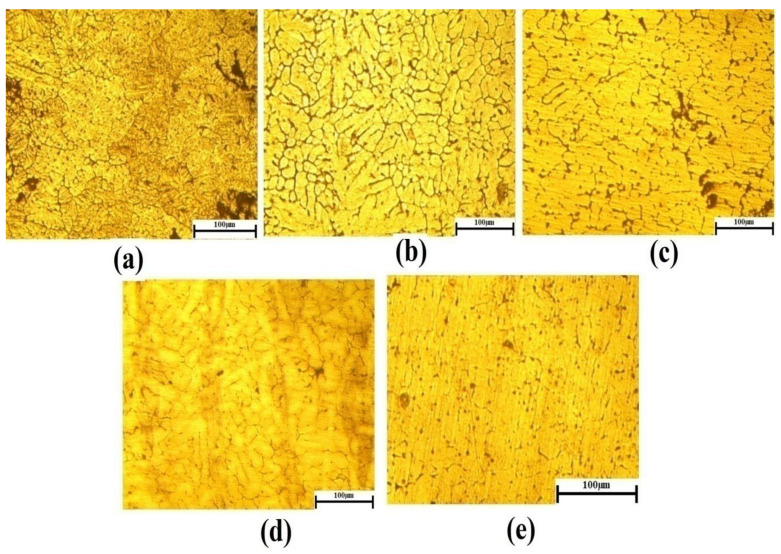
Microstructure of (**a**) non aged and aged at (**b**) 150 °C, (**c**) 250 °C, (**d**) 350 °C and (**e**) 450 °C specimen.

Figure 5 shows that the universal tensile strength of the Al7075-WC composite specimens aged at 250 °C is higher than that of the non-aged and other aged composites, due to the gradual increase in Al and Mg intermetallic compounds occupied in the grain boundary, which resists the dislocation boundary and this results in an increase in the tensile strength of the composite. In contrast, the amount of formation of Al_2_Cu, Mg_2_Si and MgZn_2_ intermetallic phases are increased when the composite is aged at 150 °C and 250 °C, as shown in Figure 4b,c, with fine grains and this could represent a marginal enhancement in the tensile strength of the composite [53]. This observation was reported as the ageing temperature is high enough to melt the Al and Mg intermetallic phases and form coarse grain, resulting in a drop in the tensile strength of the composite at 350 °C and 450 °C. In addition, as compared to the composites aged at 350 °C, there is a considerable increase in the tensile strength of the composites aged at 450 °C, which could be due to the formation of the tungsten aluminide intermetallic phase (Al_5_W), as shown in Figure 4e. Table 4 depicts the tensile strength of the Al7075-WC composites. The hardness of the composites was evaluated by the Brinell hardness setup. The composite surface was completely cleaned by the killer agent to achieve maximum accuracy and three readings were taken at different locations of the composite surface. Table 4 shows the average hardness values, and it can be observed that the Brinell hardness value of the Al7075-Tungsten carbide composite increased significantly when the ageing temperature increased, as shown in Figure 6b. The result shows that the peak hardness value of the composite was reached when the Al7075-WC composite was aged at the temperature 250 °C and this is due to the increase in the formation of Al and Mg intermetallic compounds and resistance offered by the hard phase of tungsten carbide present in the composite. These results are compatible with the work of Ridvan Yamanoglu et al. [54]. It has been observed that beyond the temperature of 250 °C, there is a decrease in the hardness value of the composite, due to the softening of the material caused by the reduction and dissolving of the intermetallic compounds, such as the precipitates of aluminum and magnesium and it can be noted as over ageing as shown in Figure 7. The formation of precipitate in the particle–matrix interfaces is appreciated by comparing the micrographs, as shown in Figure 8a–e. This reveals the formation of the precipitate during the ageing of the composite, which occupies the matrix–particle interfaces and the XRD graph analysis confirms the presence of intermetallic compounds during ageing of the Al7075-WC composites. S. B. Hassan et al. [55] reported that, regardless of the ageing period, Al-Si-Fe/SiC particle composites aged at different temperatures of 100, 200, and 300 °C showed similar behavior.

It was observed that the hardness values of the composites were increased, compared with the composite aged at 350 °C and maximum impact energy of the Al7075-WC composite was reached when the composite was aged at 450 °C, as compared with all the other Al7075-WC composite specimens, due to the formation of the hard intermetallic phase of tungsten aluminide (Al_5_W) with its higher toughness, as shown in Figure 6c. This was confirmed through XRD analysis, as shown in Figure 4e.

The tensile strength, Brinell hardness number, and impact energy of the age-hardened composites at 250 °C were increased by 37.1%, 50.5%, and 92.2%, respectively, compared to non-aged Al7075-WC composites. As the Al7075-tungsten carbide composite specimens were aged at 350 °C, their tensile strength, nominal hardness, and impact energy all decreased by 30.86%, 4.7%, and 24.9%, respectively, when compared to the composite aged at 250 °C. The mechanical properties of the Al7075-WC composite reached peak values at the age-hardening temperature region of 150 °C to 250 °C and decreased at the age-hardened temperature region of 250 °C to 350 °C. The maximum tensile strength and Brinell hardness were found in the specimen aged at 250 °C, and maximum impact energy was found when the composite was aged at 450 °C, due to the presence of hard tungsten aluminide (Al_5_W) precipitate, which leads to an increase in the toughness of the composite.

### 3.4. Fractography Study

The Al7075-WC composite specimens, after the tensile test, were subjected to SEM examination for analyzing the fracture mechanism. The non-aged composite material shearing took place due to the brittle fracture with the presence of huge matrix cracks, as shown in Figure 7a, which reduces the tensile strength and fractures occur as a result. The dimples present on the fractured surface of the Al7075-WC composites subjected to the tensile test indicate ductile fracture. Further analysis of the fracture mechanism shows the presence of voids, which leads to the initiation of cracks in the composite. The voids might have occurred in the composite during manufacturing by air and other gases may have been locked inside the cast composite when cooling. The microstructure analysis of the Al7075-WC composites reveals that the propagation of size of the cracks in the matrix is decreased, when the composite aged at 150 °C for 2 h and the tensile strength increased. as compared to the non-aged Al7075-WC composite shown in Figure 7b. When the sample was aged at 250 °C, the formation of dimples, small voids, nature of brittle fracture, and reduced matrix crack, as shown in Figure 7c, maximized the tensile strength and reduced the strain rate, due to the reduced matrix crack and strong interfacial bonding between the matrix and tungsten carbide particles, as compared to the non-aged Al7075-WC composite. In addition, as observed in the XRD graph analysis, the development of intermetallic phases increased during ageing at 250 °C, reducing voids and resisting crack propagation, due to the increasing precipitate formation in the grain boundary and as a result, the strength of the composite increased. As shown in Figure 7d, the reinforcement particle pulled out from the matrix due to the decreasing interfacial bonding strength between the reinforcement and aluminum matrix, which resulted in a significant increase in the strain rate in the composite, which leads to a decrease in the tensile strength. So, the stress strain curve shifts to the right side, as observed in Figure 5. In Figure 7e, when the composite aged at 450 °C, the strain rate was reduced due to the formation of tungsten aluminide (Al_5_W) and the stress–strain curve shifted from right to left without much increase in tensile strength, as compared to the composite aged at 350 °C.

### 3.5. Surface Topography Analysis

Figure 8a–e shows an optical microscopic image of the non-aged and age-hardened Al7075-WC composite. Figure 8a reveals the presence of spheroidal WC, dendrite arm spacing, and silicon particles in the non-aged composite. During age hardening, undissolved secondary phases were present in the grain boundaries due to its stability, even at higher temperatures. Figure 8b,c show that a greater amount of secondary phases were formed, such as Al_2_Cu, Mg_2_Si and MgZn_2,_ at the ageing temperature of 150 °C and 250 °C with fine grains and alloying elements of the composite form intermetallic compounds on the grain boundaries of the composite, which leads to an increase in the strength properties of the composites, as compared with the non-aged composite. This result is in line with what Farajollahi et al. [56] reported, meaning that the presence of the nickel aluminide structure increased the hardness, strength, and toughness after homogenization treatment and Zhang et al.’s work also supports this [57]. For the composite aged at 350 °C, Figure 8d reveals that the dissolving of Al and Mg precipitates leads to the formation of a coarse grain, which decreases the strength of the composite due to the higher dislocation movement offered by AMMCs and the secondary phase of tungsten aluminide presences in the composite during age hardening at 450 °C, which leads to the increased strength of the Al7075-WC composite, as compared to the composite aged at 350 °C due to high stability of Al_5_W, which is analyzed by EDX and the results are plotted in Figure 8e.

### 3.6. Wear Behavior of Composites

**Figure 9:**
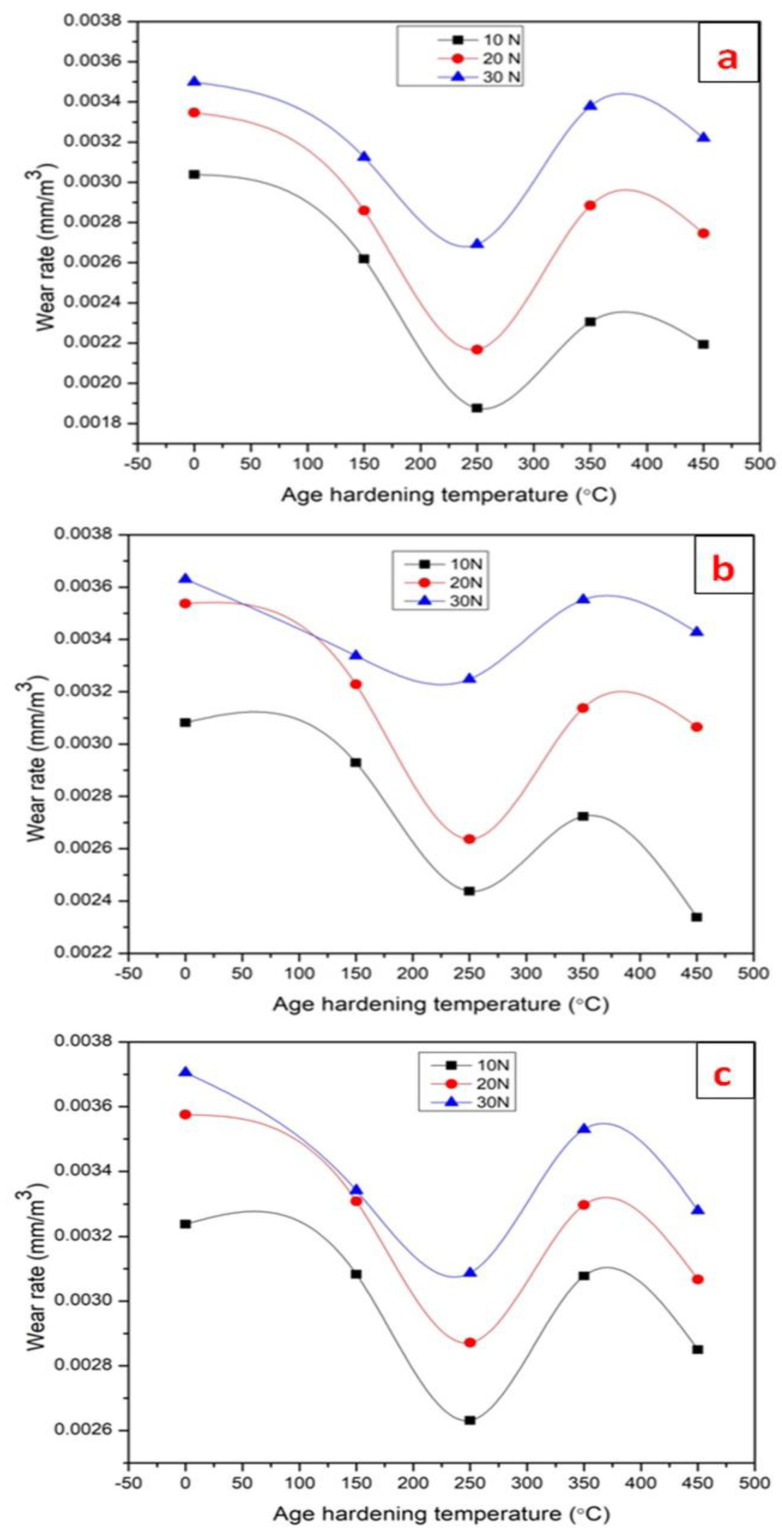
Wear rate of Al7075-WC composite with sliding distance of (**a**) 1000 m, (**b**) 1500 m; (**c**) 2000 m.

The essential wear parameters were discovered as load, sliding distance, and age-hardening temperature, and they are adjusted in three levels, as given in Table 5. The age-hardening temperatures are selected based on the mechanical properties, exhibited by the composites from the above observations. Based on the selected input parameters, such as load, sliding distance, and age-hardening temperature, the experimental work was conducted and the wear rate (output response) of the specimen was found. As shown in Figure 9a–c, the wear rate of the Al7075-WC composite materials was shown as a function of the various ageing temperatures at varying loads and sliding distances. The increased hardness value in the composite material due to the formation of secondary phases in the age-hardened composite results in enhanced wear resistance.

The wear graph of the composite aged at 150 °C and 250 °C shows an improvement in wear resistance when compared to the non-aged composite. This is due to the formation of fine grains of aluminum and magnesium precipitates in the composite, which leads to the composite resisting plastic deformation under varying loads and sliding distance. When compared to other aged composites, material removal from aluminum 7075-tungsten carbide composites aged at 350 °C was higher. This result shows that the dissolving of the precipitate leads to increased plastic deformation, whereas there is a marginal improvement in wear resistance when the composite aged at 450 °C, due to the formation of Al_5_W precipitate in the composite. Figure 9a shows that for all the loads (10 N, 20 N and 30 N) with a sliding distance of 1000m, the wear rate of the Al7075-tungsten carbide composite aged at 250 °C is 0.0018763, 0.0021674 and 0.0026912, respectively and the wear resistance is improved by 38.27%, 35.23%, and 23.08% over the non-aged Al7075-WC composite.

Figure 9b shows that for all the loads (10 N, 20 N and 30 N) with a sliding distance of 1500 m, the wear rate of the Al7075-WC composite aged at 250 °C is 0.0024377, 0.0026368 and 0.0032481 for the applied loads, respectively. The percentage increase in wear resistance compared with the non-aged Al7075-WC composite is around 20.91%, 25.46%, and 10.53%, respectively, and when the composite was subjected to age hardening, the wear resistance of the composite increased. Figure 9c shows that for all the loads (10 N, 20 N and 30 N) with a sliding distance of 2000 m, the wear rate of the Al7075-WC composite aged at 250 °C is 0.0026309, 0.0028714 and 0.0030870, respectively. The percentage of improvement in wear resistance as compared to the non-aged Al7075-WC composite is around 18.75%, 19.69% and 16.19%, respectively. As shown in Figure 9a–c, it was found that composites aged at 250 °C possess higher wear resistance for all wear parameters of load and sliding distance, as compared to non-aged and aged composites. Based on the experimental results, it was concluded that the wear rate of the Al7075-WC composite increased while increasing the load and sliding distance [40].

### 3.7. Analysis of Wear Mechanism

**Figure 10:**
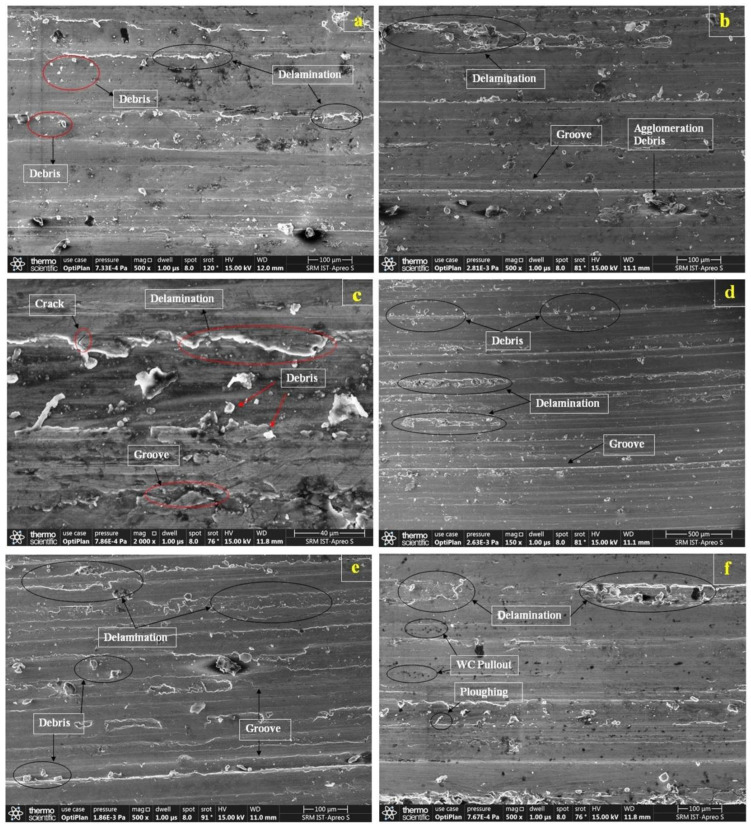
SEM micrographs of worn surfaces of Al7075-WC composites with sliding distance of 2000 m: (**a**) non-aged at 10 N; (**b**) non-aged at 20 N; (**c**) non-aged at 30 N; (**d**) 250 °C aged at 10 N; (**e**) 250 °C aged at 20 N; (**f**) 250 °C aged at 30 N.

The specimens that exhibit high and low wear resistance under higher loads and sliding distances were selected for this study in order to examine the wear mechanism of the Al7075-WC composite. The composite samples of non-aged and aged composites at 250 °C, with applied loads of 10 N, 20 N and 30 N and with the sliding distance of 2000 m were taken for the analysis of the wear mechanism. In the worn surface analysis, the wear of the composite predominantly occurred by the action of abrasion and delamination. It is observed that delamination occurred along the sliding direction on the non-aged composite, indicating higher wear loss, as shown in Figure 10a–c. The delamination area of the specimen depends on the region that undergoes crack propagation and plastic deformation that results in deep irregular grooves; more material is removed from the non-aged composite surface while increasing the applied loads and sliding distance. In addition, debris interacts with the counter surface and forms grooves on the surface of a soft material, which causes increased delamination in the composite sample. The composite aged at 250 °C shows the minimum delaminated area, as compared to the non-aged composite. As shown in Figure 10d–f, for the composite aged at 250 °C, it was observed that less debris occurred due to higher amounts of Al and Mg precipitate that were present in the composite, which increased the hardness of the material, which reduces the debris, deep grooves and material removal from the composite during contact with the counter surface [58]. Artificial aging plays a key role in improving the wear resistance of the composites due to the formation of hard secondary phases.

**Figure 11:**
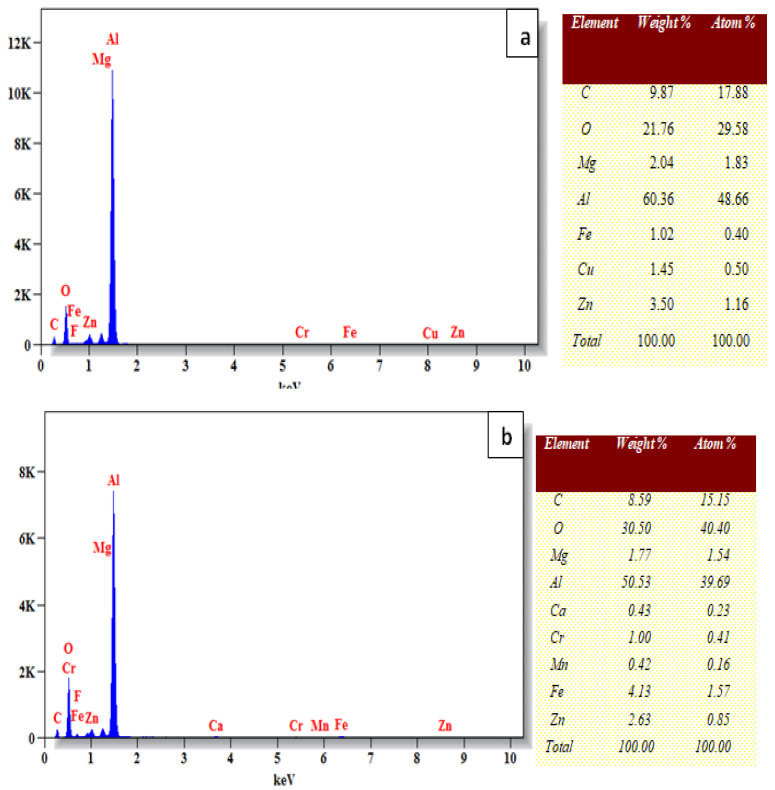
(**a**) EDS Image of composite worn surface aged at 250 °C of 30 N load at 2000 m sliding distance. (**b**) EDS Image of non-aged composite worn surface of 30 N load at 2000 m sliding distance.

As shown in Figure 11a,b, one can observe the wear surface of the Al7075-WC composites aged at 250 °C and non-aged, as studied by energy dispersive spectroscopy. The increase in sliding distance that increases the temperature of the composite pin and disc interface leads to the formation of the tribo-layer. The presence of iron (Fe) in the EDS image reveals that iron was transferred from the counter disc during the wear test of the Al 7075-WC composite, indicating that a mechanically mixed layer (MML) formed over the composite pin.

## 4. Conclusions

In the present investigation, an attempt was made to predict the age-hardening temperature with enhanced mechanical and wear properties of Al7075-WC. The impact of heat treatment on the mechanical and tribological properties of Al7075-WC was investigated in this work. The following findings were found.

- The tensile test shows that at the 250 °C age-hardening temperature, the specimen reveals higher tensile strength. The tensile strength of the 250 °C aged composite improved up to 37.1% and nominal hardness increased to 50.85%, when compared with the non-aged Al7075-WC composite, due to the presence of a higher amount of secondary phase of Al and Mg precipitates.
- The maximum impact energy was observed with the 450 °C age-hardened Al7075-WC composite with an increase of 92.2%, when compared to the non-aged composite. The tensile strength, nominal hardness and impact energy of the Al7075-WC composite decreased when the composite specimens aged at 350 °C with 30.86%, 4.7% and 24.9%, respectively, as compared to the composite aged at 250 °C, due to the dissolving of the precipitates of aluminum and magnesium compounds.
- The dimples present on the fractured surface of the Al7075-WC composites subjected to the tensile test indicated ductile fracture. The tensile fracture in the fabricated samples was a mixture of the ductile and brittle mode, according to SEM fractography investigation.
- The microstuctural analysis shows that age hardening improves the reduction in the space between the reinforcement particles of WC in the Al7075 matrix that resist the boundary dislocation of the composite, which shows fine grains at 250 °C and also spheroid WC reinforcement forms, flakes and needle-like boundaries, which increase the properties of the composites.
- The wear test was performed on a pin-on disc with various age-hardening temperatures of the Al7075-WC composites at a load of 10 N, 20 N and 30 N with a sliding distance of 1000 m, 1500 m and 2000 m. The better wear resistance was observed in the 250 °C age-hardened composite, as compared to the non-aged and aged at 150 °C, 350 °C and 450 °C composites. Because of the huge quantity of plastic deformation, more material was removed from the composite surface, resulting in increased mass loss and wear rate in the composites as the load and sliding distance increased.

## Figures and Tables

**Table 1:** Details of chemical composition of Al7075.

Material	Chemical Composition							
Al7075	**Zn**	**Cu**	**Mg**	**Mn**	**Fe**	**Si**	**Cr**	**Al**
	5.81	1.665	2.495	0.037	0.22	0.27	0.258	88.67

**Table 2:** Details of physical properties of tungsten carbide particulate.

Material	Physical Properties		
WC	**Density (gm/cc)**	**Melting Point (°C)**	**Average Size(µm)**
	15.7	2900	3–5

**Table 3:** Chemical compositional details of tungsten carbide particulate.

Material	Chemical Composition							
WC	**C**	**Fe**	**Mo**	**Si**	**Na**	**K**	**Ca**	**W**
	6.11–6.16	0.05	0.05	0.005	0.003	0.002	0.004	93.776–93.726

**Table 4:** Average tensile strength, impact energy and hardness of Al7075-WC composite.

Specification	Average Tensile Strength (MPa)	Average Brinell Hardness Number	Average Impact Energy Observed (Joules)
Non-aged	91.35	83.43	2.06
Aged at 150 °C	99.63	107.63	2.46
Aged at 250 °C	125.25	125.57	3.66
Aged at 350 °C	95.71	119.86	2.93
Aged at 450 °C	109.66	121.56	3.96

**Table 5:** Wear parameters.

Parameters	Level
Load	10 N, 20 N, 30 N
Sliding distance	1000 m, 1500 m, 2000 m
Age-hardening temperature	0 °C (non-aged), 150 °C, 250 °C, 350 °C, 450 °C
Time	15 min
Speed	600 rpm

## Data Availability

Not applicable.
